# Effects of inspiratory muscle training on exercise capacity, muscle oxygenation and strength, physical activity, and dyspnea in patients with post-COVID-19 syndrome and pulmonary involvement: a randomized controlled triple-blinded study

**DOI:** 10.1186/s12890-026-04249-4

**Published:** 2026-03-29

**Authors:** Başak Kavalcı Kol, Meral Boşnak Güçlü, Ece Baytok, Nilgün Yılmaz Demirci

**Affiliations:** 1 Institute of Health Sciences, Department of Physiotherapy and Rehabilitation, Gazi University, Ankara, Türkiye; 2 Pilot Health Coordinatorship, Geothermal Resource Rehabilitation Application and Research Center, Kırşehir Ahi Evran University, Kırşehir, Türkiye; 3 Faculty of Health Sciences, Department of Cardiopulmonary Physical Therapy and Rehabilitation, Gazi University, Ankara, Türkiye; 4 Faculty of Health Sciences, Department of Physiotherapy and Rehabilitation, Kırklareli University, Kırklareli, Türkiye; 5 Faculty of Medicine, Department of Pulmonology, Gazi University, Ankara, Türkiye

**Keywords:** COVID-19, Exercise tolerance, Muscle oxygenation, Muscle strength

## Abstract

**Background:**

Coronavirus disease 2019 (COVID-19) affects multiple organ systems, particularly the lungs, where hypoxemia may occur. Musculoskeletal problems, fatigue, and exercise intolerance frequently occur. The effects of inspiratory muscle strength training (IMST) have not been adequately investigated in patients with post-COVID-19. This study aimed to investigate the effects of IMST on functional exercise capacity, muscle oxygenation and strength, fatigue, and dyspnea in patients with post-COVID-19 syndrome and pulmonary involvement.

**Methods:**

A randomized controlled, triple-blinded study including 40 patients with post-COVID-19 syndrome and pulmonary involvement. Patients were divided into IMST and control groups. The IMST group performed IMST using a threshold inspiratory muscle trainer (POWERbreathe^®^, HaB International Ltd., UK) at 50% of MIP, while the control group performed breathing exercises for 7 days/8 weeks. Respiratory muscle strength (mouth pressure device), functional exercise capacity [6-minute walk test (6-MWT)], muscle oxygenation [near infrared spectroscopy (NIRS)], peripheral muscle strength (dynamometer), physical activity (metabolic holter), dyspnea [London Chest Activity of Daily Living Scale (LCADL)], fatigue [(Fatigue Severity Scale (FSS)] were evaluated.

**Results:**

After training, 6-MWT distance (Cohen’s *d* = 0.65), ΔSmO₂ (%), and peripheral muscle strength (Cohen’s *d* = 0.60–0.73) significantly improved in the IMST group compared to the control group (all *p* < 0.001). LCADL total score (Cohen’s *d* = 0.55, *p* = 0.008) and FSS score (Cohen’s *d* = 0.73, *p* < 0.001) also improved, while no significant changes were observed in physical activity levels, including average METs (Cohen’s *d* = 0.42, *p* = 0.301).

**Conclusions:**

IMST is a practical method for enhancing peripheral muscle strength and oxygenation, functional exercise capacity, alleviating fatigue, and improving impairments associated with long-term hypoxemia in patients with post-COVID-19 syndrome. By focusing on post-COVID-19 with clearly defined pulmonary involvement, this study provides evidence of the isolated physiological effects of IMST, an area that has remained underexplored. These findings support the integration of IMST into pulmonary rehabilitation programs for this specific patient group. Future studies with longer-term follow-up are needed to confirm the sustainability of these effects.

**Trial registry:**

Clinical Trials Registry Number: NCT05231395; Registration date: February 9, 2022.

## Introduction

Multiple organ involvement is observed in patients with coronavirus disease 2019(COVID-19), with the lungs being the most affected organ [1, 2]. The effect of lung involvement varies, with symptoms depending on disease severity in the acute, post-acute, and chronic stages. Post-COVID-19 syndrome is defined as the persistence of signs or symptoms for at least 12 weeks (≥ 3 months) after acute SARS-CoV-2 infection, without an alternative diagnosis, according to the NICE guideline [3]. The term “Long COVID” is commonly used to describe persistent symptoms following acute SARS-CoV-2 infection. The World Health Organization defines post-COVID-19 condition as symptoms that usually begin within three months of the onset of COVID-19, last for at least two months, and cannot be explained by an alternative diagnosis [4].

It has been reported that more than 60% of patients experience persistent symptoms, most commonly fatigue and dyspnea [5–8]. In the long term, patients’ exercise capacity decreases due to persistent hyperinflammation, particularly in the lungs, as well as fatigue and dyspnea. In addition, skeletal muscle abnormalities can be observed in both acute and chronic forms of COVID-19. The most common musculoskeletal problems are peripheral muscle weakness, fatigue, and exercise intolerance [9, 10]. In patients with COVID-19, systemic inflammation affects the lungs and pulmonary vasculature, and anemia reduces the oxygen-carrying capacity of the blood [11, 12]. Oxygenation of skeletal muscles is diminished due to arterial hypoxemia, which may contribute to reduced exercise capacity and physical activity levels. Thus, persistent pulmonary impairment may lead to insufficient oxygen delivery to peripheral muscles, contributing to muscle weakness and exercise intolerance. These impairments may reduce functional independence and limit activities of daily living, increasing the need for structured rehabilitation programs [13]. Given the high prevalence of persistent symptoms, post-COVID-19 syndrome also represents an increasing burden on healthcare systems [14]. However, the effectiveness of targeted interventions addressing both respiratory and peripheral muscle dysfunction in patients with post-COVID-19 syndrome and pulmonary involvement remains insufficiently established.

Inspiratory muscle training (IMT) enhances inspiratory muscle strength and reduces metaboreflex activity, thereby increasing blood flow to the peripheral muscles. This physiological situation may lead to increased peripheral muscle strength [15, 16]. The effects of IMT on peripheral muscle strength are unclear. While some studies have shown that quadriceps femoris (QF) muscle strength increases after IMT [17, 18], another study found no improvement in handgrip strength [19]. The IMT has been shown to improve oxygenation of the respiratory muscles in patients with heart failure and athletes [20, 21]. Deoxygenation of locomotor and accessory respiratory muscles was reduced during submaximal exercise after six weeks of IMT in athletes. With IMT, the oxygen demand of the respiratory muscles decreases during exercise. In contrast, the amount of oxygen delivered to the locomotor muscles increases, thereby enhancing the oxygenation of the peripheral muscles [21]. Hypoxemia may also affect the oxygenation of peripheral muscles in patients who have had COVID-19. However, the mechanisms through which IMT may influence exercise performance and muscle oxygenation in post-COVID-19 patients remain to be clarified. Improvements in inspiratory muscle strength may reduce respiratory muscle metaboreflex activity, leading to increased perfusion and oxygen delivery to locomotor muscles and thereby enhancing overall exercise tolerance. Inspiratory muscle training protocols can vary significantly in training intensity, which may affect the type of physiological adaptation achieved. Previous studies have demonstrated that loads ≥ 40% of maximal inspiratory pressure (MIP) are generally necessary to produce meaningful inspiratory muscle strength adaptations, whereas lower intensities are more often associated with endurance-focused responses [22]. In clinical populations, intensities between 40 and 60% of MIP are frequently used as they provide a balance between training effectiveness and tolerability [23, 24].

In recent years, IMT has been utilized as a treatment option in pulmonary rehabilitation, particularly for patients with inspiratory muscle weakness. The IMT applied in various lung diseases has been reported to improve respiratory muscle strength and exercise capacity, as well as reduce fatigue and dyspnea [25, 26]. There are limited studies in the literature that investigate the effects of IMT in patients with post-COVID-19 syndrome. A limited number of studies have shown that IMT increases respiratory muscle strength and exercise capacity [27–29], but its effects on muscle oxygenation, peripheral muscle strength, and symptom improvement remain uncertain. Previous studies have primarily included heterogeneous groups of COVID-19 patients with respect to disease severity, hospitalization status, and the presence or absence of objectively confirmed pulmonary involvement. Therefore, the long-term effects of IMT on exercise capacity, muscle oxygenation, muscle strength, fatigue, and dyspnea in patients with clearly defined post-COVID-19 syndrome and CT-confirmed pulmonary involvement remain unclear. To our knowledge, no randomized controlled study has evaluated the isolated effects of IMST on peripheral muscle oxygenation, functional exercise capacity, and peripheral muscle strength specifically in patients who meet standardized diagnostic criteria for post-COVID-19 syndrome and have confirmed pulmonary involvement.

The primary hypothesis was that inspiratory muscle strength training (IMST) would improve peripheral muscle oxygenation and functional exercise capacity in patients with post-COVID-19 syndrome by reducing respiratory muscle metaboreflex activity and enhancing oxygen delivery to locomotor muscles. The primary outcomes were peripheral muscle oxygenation and functional exercise capacity. Secondary outcomes included respiratory and peripheral muscle strength, physical activity level, dyspnea, and fatigue. Accordingly, this study aimed to comprehensively evaluate the effects of IMST on exercise capacity, muscle oxygenation, muscle strength, physical activity, dyspnea, and fatigue in patients with post-COVID-19 syndrome and pulmonary involvement.

## Methods

### Patients

Forty patients diagnosed with post-COVID-19 syndrome (persistent symptoms ≥ 12 weeks after acute SARS-CoV-2 infection without alternative diagnosis) were included in the study. In addition, all patients had objectively confirmed pulmonary involvement on chest CT, as evaluated by a pulmonologist. Each lung lobe was visually scored from 0 to 3, and the total CT score (0–15) was categorized as ≤ 50% (≤ 7) or > 50% (> 7) involvement [30, 31]. After the diagnosis was made by the Department of Chest Diseases, the patients were referred to the Gazi University Department of Cardiopulmonary Physiotherapy and Rehabilitation. Inclusion criteria: aged 18–75 years, with pulmonary involvement according to CT [32]. Exclusion criteria, acute respiratory tract infection, neurological, neuromuscular, orthopedic, systemic diseases, engaging in regular exercise or participation in a structured exercise program within the previous 3 months, cognitive impairment, according to the American Society of Sports Medicine, participants with absolute and relative contraindications to exercise test [33], cancer, renal or hepatic disease, aortic stenosis, complex arrhythmia, aortic aneurysm, uncontrolled hypertension, diabetes mellitus, heart failure and cardiovascular disease, CT as a result, participants with bullae in the lung close to the pleura were excluded from the study.

### Study design

The study design was randomized, controlled, and triple blind. Permission was obtained from the Gazi University Ethics Committee for the study (ID: 2022-093). The scope of the study was explained to the participants, and an informed consent form was signed. The study adhered to the ethical principles set out in the Declaration of Helsinki.

### Allocation concealment, randomization, and blinding

Participants were divided into two groups: IMST and control groups using a six-block randomization method. The codes in the random block sequences generated by a block randomization website (https://www.sealedenvelope.com) for randomization were placed in envelopes by the study director. A researcher (not a researcher in the study) defined the researcher who would conduct the training and code the envelopes according to the order in which the participants arrived. The participants, divided into groups, began their treatments. The participants’ pre- and post-training evaluations were performed by a researcher physiotherapist, and the rehabilitation follow-ups were conducted by a different researcher physiotherapist. Each patient’s assessment and training were performed at other times. The data were analyzed by another researcher. The physiotherapist administering the intervention was not engaged in outcome evaluations or data analysis. The outcome assessor did not have access to the randomization list or group allocation details. Participants were informed that both groups were receiving respiratory training interventions and were not made aware of which intervention was considered experimental. The study was conducted under a triple-blind design, in which the researchers, participants, and data analysts were all blinded to group allocation.

### Outcome measures

Static and dynamic lung volumes, as well as the carbon monoxide diffusing capacity, were measured according to the European Respiratory Society guidelines. (Vmax^®^ 220 Sensormedics Corporation, Yorba Linda, California, USA) [34]. After measurements, participants were referred to the Cardiopulmonary Rehabilitation Unit for evaluation and application of the training program. The evaluations were completed on two different days. On the first day, demographic features, clinical symptoms, respiratory and peripheral muscle strength, dyspnea during daily activities, and fatigue levels were assessed. On the second day, functional exercise capacity and QF muscle oxygenation were evaluated. By the end of the second day, evaluations were conducted using a physical activity monitor for five consecutive days. Functional exercise capacity was assessed by the six-minute walk test (6-MWT) according to American Thoracic Society (ATS) criteria [35]. The six-minute walk test distance has excellent reliability in patients with post-COVID-19 (ICC = 0.97; MDC₉₅ = 5.57%) [36]. Vital signs were monitored before, during, and after the test. The test was repeated twice on the same day, with at least a 30-minute break between the two tests. The participants were allowed to rest for 10 min before each test. The 6-MWT was conducted in a 30-meter indoor corridor under controlled room temperature (22–24 °C), with standardized verbal encouragement provided every minute in accordance with ATS guidelines. The one with the better result of the two tests was analyzed. The predicted 6-MWT distance was calculated with the reference equation of Gibbons et al. [37]. Before, during, and after the 6-MWT test, QF muscle oxygenation and total hemoglobin level were measured using the Moxy^®^ (near-infrared spectroscopy system) monitor (Moxy, Fortiori Design LLC, Minnesota, USA). The monitor measures local oxygen saturation and total hemoglobin in muscle capillaries below the motor point of the muscle [38]. The Moxy sensor was placed on the motor point of the vastus lateralis portion of the quadriceps femoris muscle, approximately one-third of the distance between the trochanter major and the lateral border of the patella [38]. For resting measurements, at least 3 min were waited after the device was inserted. Minimum (SmO_2_min) and maximum (SmO_2_max) muscle oxygen saturation, as well as minimum (THbmin) and maximum (THbmax) total hemoglobin, were measured using the device. ΔSmO₂ was calculated as the difference between the maximum and minimum SmO₂ values recorded during the 6-MWT (ΔSmO₂=SmO_2_max₂−SmO_2_min₂). The Moxy oxygen monitor is a valid and reliable device for assessing muscle oxygenation. SmO₂ reliability has been reported as good–excellent (Spearman’s ρ = 0.83–0.98; ICC = 0.77–0.99). The reproducibility of SmO₂ and related change values (ΔSmO₂) measured with the Moxy monitor has been reported as good to excellent (ICC = 0.79–0.92 [39]. For THb, test–retest reliability is also high, with a coefficient of variation ≤ 1% across all exercise intensities [38]. The Moxy Monitor was factory-calibrated according to the manufacturer’s specifications, and calibration updates were applied regularly. To minimize motion artifacts, the device was securely fixed to the skin with hypoallergenic plaster to ensure full contact with the tissue. Signal quality was verified using the device’s built-in indicator, and only data with “good” or “excellent” signal quality were included in the analysis [38].

Muscle strength of the deltoid and QF was evaluated with a handheld dynamometer (JTECH Power Track Commander, Baltimore, USA). Reference equations by Andrews and colleagues were used to calculate the percentage of peripheral muscle strength predicted [40]. The JTECH handheld dynamometer was calibrated annually according to the manufacturer’s recommendations, and the device was zeroed before each measurement session to ensure accuracy. Handheld dynamometry has been shown to provide excellent reliability for measuring quadriceps (ICC = 0.95) and deltoid (ICC = 0.90) muscle strength in healthy adults [41, 42].

Respiratory muscle strength was evaluated with an electronic mouth pressure device according to ATS and ERS criteria (Micro Medical MicroRM, England [43]. The MIP and MEP measurements have demonstrated excellent reliability, with ICC values ranging from 0.86 to 0.90 [44]. Lista-Paz et al.‘s equations were used to calculate the predicted value [45].

Multisensory metabolic holter (Sensewear Bodymedia^®^, Inc Pittsburgh, USA) was used to evaluate physical activity level. The device was placed on the patient’s non-dominant arm over the triceps brachii muscle for 5 consecutive days a week [46]. Participants’ physical activity level was classified according to the number of steps and metabolic equivalents (METs) [47, 48]. Compliance was confirmed by verifying the device’s recorded wear time. Data were included in the analysis only when wear time was ≥ 20 h per day for at least 4 consecutive days [49].

The London Chest Activities of Daily Living Scale (LCADL) was used to evaluate dyspnea occurring during activities of daily living [50, 51]. The scale comprises a total of 4 subgroups and 15 items: self-care, domestic, physical, and leisure activities (each with 4 items). Each item is scored on a scale of 0–5 points, with a total score of 75 points. When the total score increases, dependence on activities of daily living increases [51].

Participants’ fatigue severity was evaluated using the Fatigue Severity Scale (FSS). The scale determines the severity of fatigue, comprising 9 questions, and the answers to each question are scored on a 1–7 scale. The total score from the scale ranges from 9 to 63 points. A score of 36 points or above indicates severe fatigue [52].

### Intervention protocol

After the evaluations, participants in the IMST group received IMST at 50% of MIP for 8 weeks. IMST was performed using a threshold loading device (PowerBreathe^®^, IMT Technologies Ltd., UK) with adjustable inspiratory resistance. Training at 50% of MIP represents a moderate training intensity commonly used in inspiratory muscle training protocols. MIP was reassessed weekly, and the training intensity was adjusted to 50% of the updated MIP value to maintain progressive overload throughout the intervention period. The IMST was performed once a week under the supervision of a specialist physiotherapist and at home on the remaining six days. Participants performed IMST once daily for 30 min in total. Each cycle consisted of 10–15 deep inspiratory efforts followed by 3–4 controlled breathing cycles for recovery before continuing. Participants completed an IMST diary, which was then reviewed.

Participants in the control group were instructed to perform thoracic expansion exercises as a home program once daily for approximately 30 min over 8 weeks. The exercise protocol consisted of repeated thoracic expansion breathing cycles. Each cycle included 3–4 thoracic expansion breaths followed by three breathing control breaths, and this cycle was repeated until approximately 120 thoracic expansion breaths were completed. Short rest periods were allowed between cycles depending on the participants’ tolerance. Participants kept a daily exercise diary, which was reviewed during weekly telephone follow-ups to monitor adherence to the program.

### Statistical analysis

To calculate the sample size, a pilot study was conducted, including 10 participants from each group for training. In this study, it was calculated that a total of 36 participants, 18 in the IMST group and 18 in the control group, according to the 6-MWT distance, with an effect size of *d* = 1.13, α = 0.05 type I error, β = 0.05 type II error, and power 95% (G*Power (Version 3.1.9.2). For statistical analysis, the statistical analysis program ‘Statistical Package for Social Science for Windows version (SPSS) 26.0’ (SPSS Inc., Chicago, USA) was used. The suitability of the variables for normality was evaluated analytically using the Shapiro-Wilk test. Student’s t-test was used to compare variables with a normal distribution, and the Mann-Whitney U test was used to compare variables that did not comply with normality. Mean and standard deviation were given for normally distributed variables, and median and 25-75th percentiles were given for non-normally distributed variables. Categorical variables were compared with the Pearson Chi-square or Fisher’s Exact test. Intention-to-treat analysis was used to minimize bias due to missing data when evaluation parameters could not be fully collected, and missing outcome data were handled using multiple imputation. Two-way ANCOVA test was used to compare the effect of training between groups, and Bonferroni correction was applied for post hoc comparisons. The study computed power using the G*Power (Version 3.1.9.2), and effect sizes were calculated using the F test. In the statistical analysis, the margin of error was determined to be *p* < 0.05, and the probability of error between *p* = 0.05 and 0.06 was considered close to statistical significance.

## Results

**Fig. 1:**
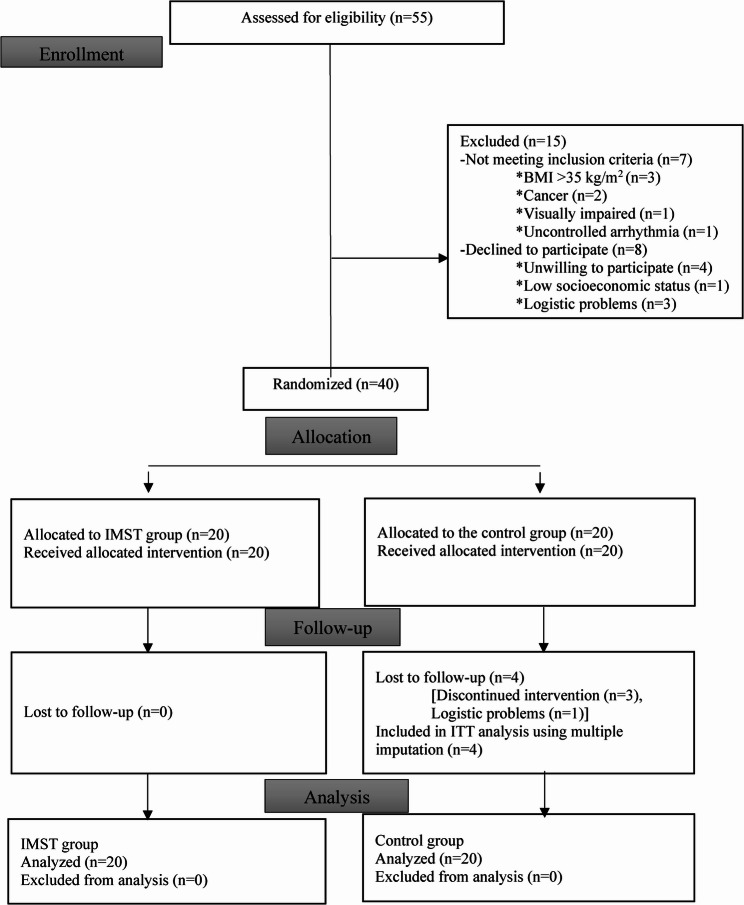
CONSORT diagram of patient flow chart Abbreviations: BMI: Body mass index, kg: kilogram, m2: square meter, IMST: inspiratory muscle strength training, CONSORT: The Consolidated Standards of Reporting of Trials

**Fig. 2:**
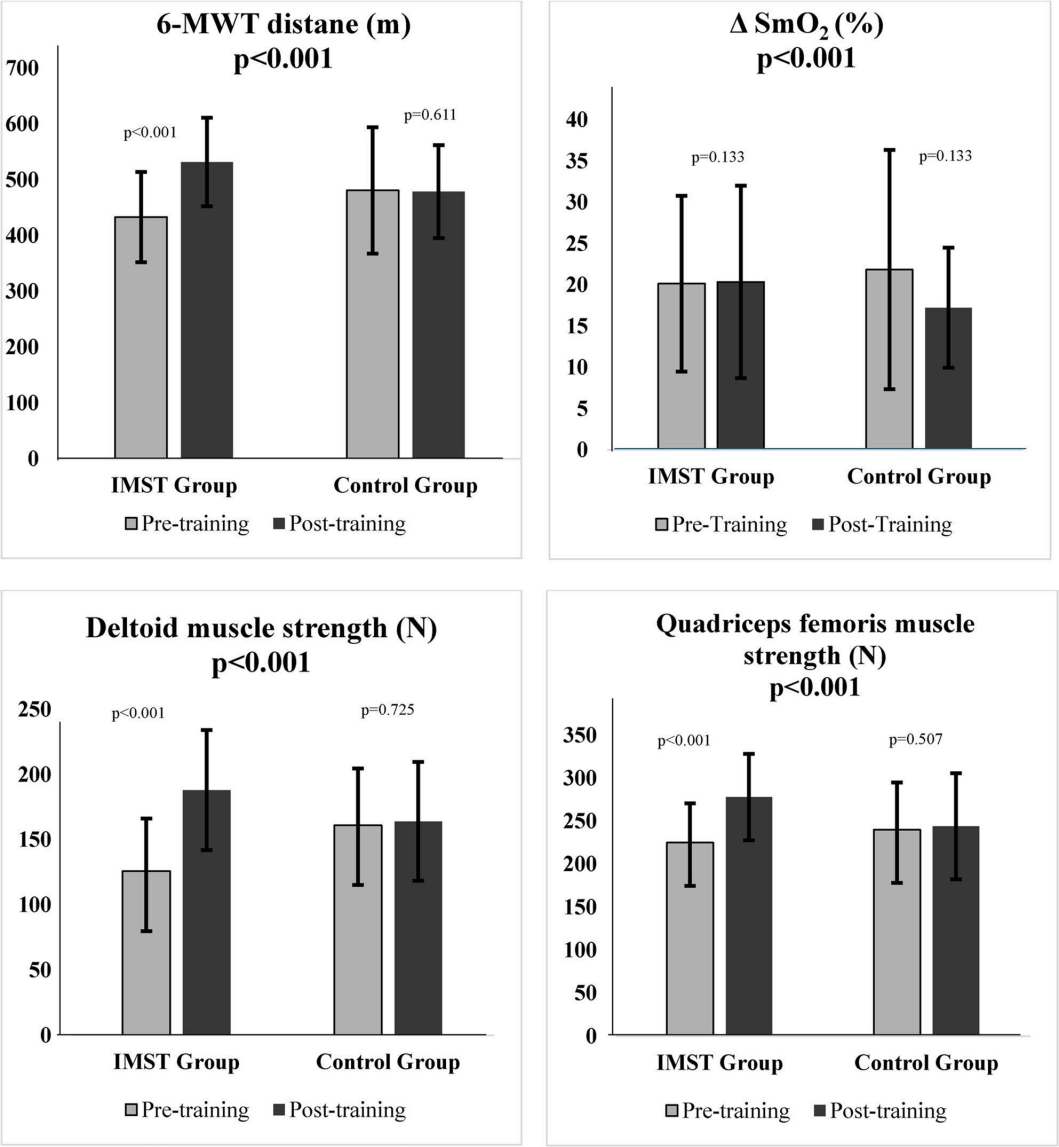
Comparison of 6-MWT distance, ΔSmO_2_, deltoid and quadriceps femoris muscle strength after IMST Abbreviations: 6-MWT: six-minute walk test, m: meter, IMST: inspiratory muscle strength, ΔSmO_2_: SmO_2_maksimum and SmO_2_minimum difference, N: Newton)

Participant flow throughout the study is presented in Fig. 1 in accordance with CONSORT guidelines. The demographic and clinical characteristics of the participants in the IMST group and the control group were statistically similar (*p* > 0.05, Table 1). Post-training, the 6-MWT distance (96.44 m, 95% CI [51.39-141.49], Cohen’s *d* = 0.65) and 6-MWT% (12.56%, 95% CI [7.94–22.40], Cohen’s *d* = 0.77) showed statistically significant increases in the IMST group compared to the control group after the intervention (*p* < 0.05, Fig. 2; Table 2). The power of the study, as indicated by the 6-MWT distance (m), was high (1-β = 98.2%). Post-training, the 6-MWT distance in the IMST group increased by an average of 98.75 m, whereas the control group decreased by 2.31 m.

**Table 1:** Comparison of demographic and clinical characteristics of the inspiratory muscle strength training group and the control group in patients with post-COVID-19 syndrome

	IMST group (*n* = 20)	Control group (*n* = 20)		
	X ± SD Median (IQR_25−75%_)	X ± SD Median (IQR_25−75%_)	Difference between means [95% CI] and medians [U]	*p*
Female (n /%); male (n /%)	10 (50%); 10 (50%)	9 (45%); 11 (55%)		0.752
Age (years)	59.75 ± 9.31	61.35 ± 6.76	1.60 (-6.81-3.61)	0.538
Height (cm)	165.90 ± 10.72	164.35 ± 10.31	1.55 (-5.18-8.28)	0.644
Weight (kg)	76.10 ± 15.10	74.40 ± 15.88	1.70 (-8.22-11.62)	0.731
Body mass index (kg/m^2^)	27.52 ± 3.93	27.43 ± 3.85	0.08 (-2.40-2.57)	0.945
Cachexia (< 18.5 kg/m^2^)	0 (0%)	0 (0%)		0.803
Normal (18.6–24.9 kg/m^2^)	6 (30%)	7 (35%)		
Overweight (25.0–29.9 kg/m^2^)	8 (40%)	6 (30%)		
Class I Obese (30.0–34.9 kg/m^2^)	6 (30%)	7 (35%)		
Hypertension	9 (45%)	10 (50%)		1.000
Coronary artery disease	4 (20%)	6 (30%)		
Diabetes mellitus	4 (20%)	4 (20%)		
Asthma	6 (30%)	1 (5%)		
COPD	2 (10%)	3 (15%)		
Heart Failure	1 (5%)	1 (5%)		
ILD	0 (0%)	1 (5%)		
Smoking (pack-years)	29.0 ± 24.64	32.39 ± 25.81	-3.39 (-23.82-17.03)	0.735
History of smoking				
Smoker (n /%)	1 (5%)	2 (10%)		0.828
Ex-smoker (n /%)	12 (60%)	11 (55%)		
Non-smoker (n /%)	7 (35%)	7 (35%)		
Time from COVID-19 diagnosis (weeks)	86.0 (56.0-126.0)	66.0 (48.0–92.0)	145.5	0.142
Lung infiltrates on CT				0.235
≤50%	18 (90%)	14 (70%)		
>50%	2 (10%)	6 (30%)		
Hospitalization (n / %)	11 (55%)	11 (55%)		1.000
Duration of hospitalization (days)	12.72 ± 11.14	9 (7–10)	-4.73 (-14.53- 5.08)	0.327
Intensive care stay (n / %)	5 (25%)	6 (30%)		0.723
Duration of intensive care stay (days)	6.60 ± 5.13	8.5 ± 6.75	-1.9 (-10.23- 6.43)	0.618
Mechanical ventilation use (n/%)	5 (25%)	3 (15%)		0.695
Duration of mechanical ventilation (days)	5.2 ± 3.7	5 ± 3.6	0.2 ( -6.36- 6.76)	0.943
Corticosteroids use (n / %)	9 (45%)	8 (40%)		0.749
Duration of corticosteroids (days)	10 (8–24)	10 (7–18)	29.5	1.000
Corticosteroid dose (mg/day)	6 (4.5–9.5)	11 (6-32.5)	19.5	0.328
FEV_1_ (L)	1.91 (1.67–2.59)	2.10 (1.70–2.68)	176	0.529
FEV_1_ (%)	91.5 (66.0-104.5)	89.0 (81.5–107.0)	173	0.478
FVC (L)	2.58 (2.12–3.18)	2.80 (2.21–3.61)	184.5	0.678
FVC (%)	101.5 (72.5–109)	93.5 (90-110.5)	189	0.779
FEV_1_/FVC	75.72 ± 12.07	77.58 ± 8.08	-1.86 (-8.44-4.71)	0.569
PEF (L)	5.76 ± 2.0	6.09 ± 2.15	-0.33 (-1.66-1.0)	0.621
PEF (%)	87.25 ± 23.98	91.20 ± 22.21	-3.95 (-18.74-10.84)	0.592
FEF _25-75%_ (L)	2.02 ± 1.13	2.10 ± 1.27	-0.07 (-0.84-0.70)	0.846
FEF_25-75%_ (%)	63.10 ± 30.34	66.75 ± 31.83	-3.65 (-23.56-16.26)	0.713
DLCO (L)	5.28 (4.31–7.39)	5.42 (4.30–6.76)	194.5	0.883
DLCO (%)	68.0 (56.5–95.0)	70.50 (65.0-85.5)	196	0.925
TLC (L)	3.65 (3.12–4.87)	4.06 (3.19–4.72)	198	0.968
TLC (%)	82.0 (58.0–98.0)	84.0 (70.0-93.5)	190.5	0.799
FRC (L)	2.34 (2.02-3.0)	2.29 (1.82–2.65)	178	0.565
FRC (%)	86.25 ± 24.45	80.85 ± 18.79	5.40 (-8.56-19.36)	0.438
RV (L)	1.89 ± 0.48	1.76 ± 0.41	0.01 (-0.28-0.31)	0.335
RV (%)	95.65 ± 23.49	88.25 ± 22.14	1.25 (-13.58-15.98)	0.312
RV/TLC (%)	43.0 (39.0-51.5)	43.0 (39.0–49.0)	183.5	0.857
Average METs (METs/day) *According to average METs*	1.20 (1.20–1.35)	1.35 (1.15–1.45)	157	0.238
• Inactive	18 (90%)	15 (75%)		0.204
• Low active	2 (10%)	5 (25%)		
Number of steps (steps/day)	6047.60 ± 3237.33	4804.20 ± 2443.27	1243.4 (-592.55-3079.35)	0.178
*According to the number of steps*				
• Inactive (n/%)	9 (45%)	12 (60%)		0.673
• Low active (n/%)	3 (15%)	3 (15%)		
• Somewhat active (n/%)	5 (25%)	4 (20%)		
• Active (n/%)	3 (15%)	1 (5%)		
• Highly active (n/%)	0 (0%)	0 (0%)		

*IMST* inspiratory muscle strength training, *cm* centimeter, *kg* kilogram, *kg/m*^*2*^ kilogram/meter square, *n* frequency, *%* percentage, *m* meter, *PCFS* post COVID-19 functional status scale, *MET* metabolic equivalent, *mg* milligram, *FEV*_*1*_ forced expiratory volume in one second, *FVC* forced vital capacity, *PEF* peak expiratory flow, *FEF*_*25–75%*_ forced expiratory flow from 25 to 75%, *DLCO* pulmonary diffusion capacity, *TLC* total lung capacity, *FRC* functional residual capacity, *RV* residual volume, *L* liter, *%* percentage, *CT* computed tomography, *COPD* chronic obstructive pulmonary disease, *ILD* interstitial lung disease, *IQR* interquartile range, *X±SD* mean ± standard deviation

*p* < 0.05

**Table 2:** Effects of inspiratory muscle strength training on functional exercise capacity in patients with post-COVID-19 syndrome

	IMST Group (*n* = 20)			Control Group (*n* = 20)			
	Pre-training	Post- training	Group difference	Pre-training	Post-training	Group difference	Treatment effect
	X ± SD	X ± SD	*p*	X ± SD	X ± SD	*p*	*p*
6-MWT (m)	433.10 ± 81.91	531.85 ± 79.33	< 0.001*	480.92 ± 113.15	478.61 ± 83.67	0.611	< 0.001*
6-MWT (%)	66.28 ± 11.46	81.52 ± 11.56	< 0.001*	73.65 ± 15.92	73.59 ± 11.68	0.464	< 0.001*
Resting HR (beats/min)	77.20 ± 12.51	73.60 ± 13.91	0.090	79.25 ± 13.84	79.82 ± 11.2	0.664	0.133
∆ HR (beats/min)	35.80 ± 12.59	43.30 ± 11.41	0.024*	38.05 ± 14.41	35.79 ± 13.78	0.626	0.051*
Peak HR (%)	72.89 ± 8.52	75.52 ± 9.85	0.243	75.52 ± 7.21	75.26 ± 7.69	0.859	0.483
Resting SBP (mmHg)	112.5 ± 10.07	111.75 ± 12.9	0.334	119.0 ± 11.65	120.87 ± 16.05	0.196	0.120
∆ SBP (mmHg)	18.5 ± 8.29	25.75 ± 18.01	0.025*	17.0 ± 12.61	11.45 ± 11.13	0.076	0.006*
Resting DBP (mmHg)	74.0 ± 9.54	74.5 ± 7.41	0.785	76.0 ± 12.31	79.97 ± 10.04	0.016*	0.057*
Δ DBP (mmHg)	6.25 ± 10.75	7.0 ± 10.43	0.213	1.25 ± 10.75	1.71 ± 9.55	0.469	0.171
Resting SpO_2_ (%)	95.25 ± 2.17	95.6 ± 2.16	0.243	94.0 ± 3.21	93.77 ± 3.32	0.401	0.164
∆ SpO_2_ (%)	-2.8 ± 2.75	-2.45 ± 3.38	0.531	-3.05 ± 4.82	-2.36 ± 4.26	0.296	0.764
Resting BF (breath/min)	20.1 ± 3.52	21.2 ± 4.07	0.316	20.4 ± 2.56	21.16 ± 4.78	0.383	0.925
∆ BF (breath/min)	12.10 ± 4.18	7.9 ± 5.1	0.001*	11.50 ± 3.66	10.0 ± 4.78	0.126	0.127
Resting dyspnea (M. Borg Scale 0–10 points)	0.55 ± 0.89	0.25 ± 0.72	0.134	0.2 ± 0.62	0 ± 0	0.004*	0.290
Δ Dyspnea (M. Borg Scale 0–10 points)	2.95 ± 1.73	1.45 ± 1.32	0.010*	1.5 ± 1.54	1.79 ± 1.58	0.397	0.219
Resting fatigue (M. Borg Scale 0–10 points)	0.43 ± 0.88	0.35 ± 0.79	0.999	0.2 ± 0.69	0.38 ± 0.74	0.519	0.649
Δ Fatigue (M. Borg Scale 0–10 points)	2.43 ± 1.63	1.13 ± 1.47	0.217	0.8 ± 1.32	1.21 ± 1.42	0.204	0.981
Resting QF fatigue (M. Borg Scale 0–10 points)	0.35 ± 0.67	0.22 ± 0.53	0.602	0.1 ± 0.45	0.24 ± 0.52	0.491	0.399
Δ QF fatigue (M. Borg Scale 0–10 points)	1.65 ± 1.66	1.27 ± 1.23	0.584	1.05 ± 1.36	1.34 ± 1.27	0.808	0.580

*IMST* inspiratory muscle strength training, *6-MWT* six minute walk tets, *HR* heart rate, *SBP* systolic blood pressure, *DBP* diastolic blood pressure, *BF* breathing frequency, *M. Borg Scale* Modified Borg Scale, *QF* quadriceps femoris

SpO_2_: oxygen saturation, m: meter, min: minutes, mmHg: millimeters of mercury, %: percentages, Δ: Difference between post-test and pre-test value, X**±**SD: mean ± standard deviation

*Statistically significant p-values (*p* < 0.05)

Post-training, QF muscle ΔSmO_2_% and ΔSmO_2_average% of the control group during the 6-MWT decreased significantly compared to the IMST group (*p* < 0.001, Fig. 2; Table 3).In the IMST group, SmO_2_rest% and SmO_2_min% increased significantly within the group, while ΔTHb (g/dl) decreased (*p* < 0.05). Meanwhile, ΔTHb (g/dl) decreased in the control group (*p* = 0.001, Table 3).

**Table 3:** Effects of inspiratory muscle strength training on quadriceps femoris muscle oxygenation and total hemoglobin levels in patients with post-COVID-19 syndrome

	IMST Group (*n* = 20)			Control Group (*n* = 20)			
	Pre-training	Post-training	Group difference	Pre-training	Post-training	Group Difference	Treatment effect
	X ± SD	X ± SD	*p*	X ± SD	X ± SD	*p*	*p*
SmO_2_resting (%)	49.15 ± 16.14	55.65 ± 15.88	0.031*	47.35 ± 15.55	47.81 ± 14.69	0.994	0.120
SmO_2_min (%)	25.35 ± 17.44	31.4 ± 14.35	0.041*	26.35 ± 19.63	28.57 ± 17.21	0.380	0.391
SmO_2_max (%)	45.50 ± 13.02	51.75 ± 16.18	0.078	48.20 ± 18.63	45.86 ± 15.62	0.588	0.105
ΔSmO_2_ (%)	20.15 ± 10.65	20.35 ± 11.65	0.133	21.85 ± 14.49	17.22 ± 7.28	0.133	< 0.001*
SmO_2_average-min (%)	26.0 ± 17.49	31.4 ± 14.78	0.091	27.70 ± 19.68	30.09 ± 17.42	0.339	0.523
SmO_2_average-maks (%)	46.45 ± 14.37	51.65 ± 15.94	0.143	48.15 ± 18.05	45.24 ± 15.15	0.464	0.123
ΔSmO_2_average (%)	20.45 ± 11.08	20.25 ± 12.12	0.101	20.45 ± 14.14	15.73 ± 7.44	0.101	**< **0.001*
SmO_2_recovery (%)	45.20 ± 18.23	49.75 ± 12.46	0.111	46.15 ± 20.60	45.0 ± 16.65	0.736	0.172
SmO_2_recovery-average (%)	44.50 ± 18.34	48.9 ± 12.62	0.120	45.45 ± 20.22	43.54 ± 16.67	0.527	0.123
THbresting (g/dl)	12.26 ± 0.43	12.19 ± 0.37	0.368	12.26 ± 0.45	12.29 ± 0.38	0.749	0.389
THbmin (g/dl)	11.79 ± 0.42	11.86 ± 0.35	0.402	11.83 ± 0.38	11.88 ± 0.26	0.367	0.963
Thbmax(g/dl)	12.18 ± 0.4	12.15 ± 0.33	0.426	12.24 ± 0.39	12.13 ± 0.27	0.203	0.730
ΔTHb (g/dl)	0.39 ± 0.27	0.29 ± 0.16	0.003*	0.4 ± 0.33	0.27 ± 0.13	0.001*	0.692
THbrecovery (g/dl)	12.08 ± 0.5	12.07 ± 0.33	0.610	12.17 ± 0.42	12.12 ± 0.4	0.708	0.924

*IMST *inspiratory muscle strength training, *SmO*_*2*_ Muscle oxygen saturation, *THb* Total hemoglobin

%: percentage, min: minimum, max: maximum, g gram: dl: deciliter Δ: Difference between post-test and pre-test values, X ± SD: mean ± standard deviation

*Statistically significant p-values (*p* < 0.05)

Post-training, the dominant QF (48.69 N, 95% CI [32.43–64.09] N, Cohen’s *d* = 0.60) and deltoid muscle strength (68.71 N, 95% CI [39.62–79.15] N, Cohen’s *d* = 0.73) of the IMST group increased significantly compared to the control group (*p* < 0.05, Fig. 2), while there was no difference in non-dominant QF muscle strength (*p* = 0.119, Table 4).

**Table 4:** Effects of inspiratory muscle strength training on respiratory and peripheral muscle strength, physical activity level, dyspnea during daily living activity, and fatigue severity in patients with post-COVID-19 syndrome

		IMST Group (*n* = 20)		Control Group (*n* = 20)			
	Pre-training	Post-training	Group Difference	Pre-training	Post-training	Group Difference	Treatment effect
	X ± SD	X ± SD	*p*	X ± SD	X ± SD	*p*	*p*
MIP (cmH_2_O)	76.80 ± 25.89	126.05 ± 32.9	**<** 0.001*	92.75 ± 29.79	88.4 ± 28.62	0.307	0.003*
MIP (%)	68.76 ± 20.60	112.87 ± 24.41	< 0.001*	81.84 ± 22.89	78.31 ± 21.58	0.402	**< **0.001*
MEP (cmH_2_O)	111.15 ± 35.04	155.80 ± 32.77	**<** 0.001*	129.35 ± 40.12	124.1 ± 36.75	0.497	< 0.001*
MEP (%)	62.59 ± 16.22	88.55 ± 16.96	**<** 0.001*	71.35 ± 16.68	68.21 ± 15.27	0.510	**<** 0.001*
Deltoid (D) (N)	126.95 ± 40.31	188.0 ± 46.07	**< **0.001*	161.74 ± 43.63	154.69 ± 45.62	0.725	**< **0.001*
Deltoid (D) %	72.69 ± 20.16	107.42 ± 23.03	**<** 0.001*	93.44 ± 19.61	88.8 ± 23.0	0.680	< 0.001*
Deltoid (ND) (N)	122.90 ± 34.62	178.5 ± 45.01	**< **0.001*	154.26 ± 49.68	152.78 ± 42.8	0.664	**<** 0.001*
Deltoid (ND) %	75.47 ± 17.56	109.6 ± 24.73	**< **0.001*	92.63 ± 17.07	93.4 ± 22.64	0.588	**<** 0.001*
Quadriceps femoris (D) (N)	225.30 ± 45.61	278.4 ± 50.16	**< **0.001*	240.30 ± 54.97	244.71 ± 61.65	0.507	**< **0.001*
Quadriceps femoris (D) %	65.15 ± 13.29	80.6 ± 16.11	< 0.001*	70.60 ± 12.38	71.34 ± 9.99	0.530	< 0.001*
Quadriceps femoris (ND) (N)	230.20 ± 51.47	267.05 ± 43.1	**<** 0.001*	248.75 ± 48.93	259.55 ± 54.3	0.104	0.119
Quadriceps femoris (ND) %	67.13 ± 13.90	78.85 ± 17.92	< 0.001*	73.82 ± 12.53	76.68 ± 15.07	0.237	0.069
Total energy expenditure (joules/day)	9317.70 ± 1730.27	9336.07 ± 1603.92	0.941	9635.90 ± 2021.63	9859.98 ± 1913.49	0.253	0.388
Active energy expenditure (joules/day)	1109.40 ± 1136.33	1112.69 ± 582.32	0.704	1254.20 ± 976.46	1253.97 ± 703.99	0.688	0.581
Physical activity duration (> 3MET) (min/day)	56.55 ± 44.18	55.65 ± 32.44	0.590	64.35 ± 49.0	59.63 ± 32.29	0.746	0.879
Average METs (METs/day)	1.27 ± 0.21	1.28 ± 0.17	0.802	1.32 ± 0.18	1.34 ± 0.11	0.224	0.301
Number of steps (steps/day)	6047.60 ± 3237.33	6123.14 ± 2823.65	0.463	4804.20 ± 2443.26	5489.9 ± 2448.74	0.450	0.988
Sleep duration (min/day)	394.25 ± 888.35	376.22 ± 83.01	0.316	388.1 ± 114.67	355.22 ± 86.75	0.038*	0.429
Lying down (min/day)	487.9 ± 79.22	467.18 ± 93.52	0.293	483.25 ± 111.31	454.56 ± 88.42	0.108	0.684
LCADL self-care (0–20 points)	6.15 ± 2.11	4.85 ± 1.39	0.023*	5.35 ± 2.06	5.27 ± 2.76	0.508	0.240
LCADL domestic activities (0–30 points)	7.95 ± 6.75	4.3 ± 2.87	0.001*	5.35 ± 4.21	6.15 ± 4.63	0.947	0.017*
LCADL physical activities (0–10 points)	6.4 ± 1.57	3.4 ± 1,6	**<** 0.001*	4.5 ± 1.73	4.79 ± 1.42	0.236	0.002*
LCADL leisure activities (0–15 points)	5.05 ± 2.11	3.7 ± 1.03	< 0.001*	4.55 ± 1.76	3.83 ± 1.52	0.002*	0.445
LCADL total score (0–75 points)	24.55 ± 8.75	16.25 ± 4.68	**<** 0.001*	19.9 ± 8.17	19.93 ± 8.16	0.321	0.008*
Fatigue Severity Scale (9–63 points)	48.15 ± 11.19	33.0 ± 13.83	< 0.001*	43.8 ± 14.27	42.93 ± 13.49	0.516	**< **0.001*

*IMST* inspiratory muscle strength training, *MIP* maximal inspiratory pressure, *MEP* maximal expiratory pressure,  *D* dominant, *ND* non-dominant, *LCADL* London Chest Activity of Daily Living Scale, *min* minutes, *MET* metabolic equivalent

cmH_2_O centimeter water pressure, % percentage, equivalent X **±** SD mean ± standard deviation

*Statistically significant p-values (*p* < 0.05)

The MIP (52.64 cmH_2_O, 95% CI [29.73–60.07] cmH_2_O, Cohen’s *d* = 1.24), MEP (45.59 cmH_2_O, 95%CI [21.09–57.7] cmH_2_O, Cohen’s *d* = 0.91), MIP%, and MEP% significantly increased in the IMST group compared to the control group after training (*p* < 0.05, Table 4). The mean number of attended sessions was 54.5 ± 13.24 in the IMST group and 44.39 ± 21.71 in the control group, with no statistically significant difference between groups (*p* = 0.085).

There were no significant differences in total energy expenditure, active energy expenditure, physical activity time, average METs (0.07, 95% CI [–0.11 to 0.25] METs, Cohen’s *d* = 0.42), number of steps, sleep duration, or lying down of the IMST and control groups’ post-training (*p* > 0.05, Table 4). For average METs, the observed effect size was small (Cohen’s *d* = 0.42) and the post-hoc power was 25%.

Post-training, the LCADL subscale, domestic physical activity, and total score (5.69 points, 95% CI: 3.07–13.46, Cohen’s *d* = 0.55) of the IMST group significantly decreased compared to the control group (*p* < 0.05, Table 4). Post-training, the FSS score (13.91 points, 95%CI [9.43–22.57] points, Cohen’s *d* = 0.73) of the IMST group decreased statistically significantly compared to the control group (*p* < 0.001, Table 4).

## Discussion

As a result of the current study, it was found that IMST applied for eight weeks in patients with post-COVID-19 syndrome and CT-confirmed pulmonary involvement increased functional exercise capacity, muscle oxygenation, and upper and lower extremity muscle strength, inspiratory and expiratory muscle strength, and reduced the severity of dyspnea during daily living activities and fatigue. IMST did not affect physical activity levels. In fact, IMST improved dyspnea occurring during activities of daily living and fatigue severity. It is well established that improvements in respiratory muscle function led to a reduction in dyspnea [53]. IMST is an effective training method for reducing perceived dyspnea and fatigue, the most common long-term symptoms. These findings suggest that IMST may improve both respiratory and peripheral muscle function in patients with post-COVID-19 syndrome and CT-confirmed pulmonary involvement.

There are a limited number of studies in the literature investigating the effects of IMT on functional exercise capacity in patients with COVID-19 [27, 29]. Abodonya et al. applied IMT (50% of MIP, 30 min/day, 5 days/2 weeks) and breathing exercises (2 times/day, 7 days/2 weeks) immediately after weaning patients recovering from COVID-19 from mechanical ventilation. The IMT increased patients’ functional exercise capacity by 38.8 m, while the 6-MWT distance increased by 43.9 m in the training group and by 5.1 m in the control group [29]. Del Corral et al. applied IMT (50% of MIP for 40 min/day, 6 days/week, 8 weeks) to the control group, along with unloaded training. They evaluated patients’ exercise capacity using the Ruffier test. There was no increase in exercise capacity after the training [27]. The Ruffier test is an exercise test in which patients perform 30 squats in 45 s, used to determine cardiorespiratory endurance based on pre-test, post-test, and recovery heart rate responses [54]. This test may not have fully reflected the patients’ increase in functional exercise capacity because it does not include walking activities that reflect daily living. In a recently published systematic review, it was shown that IMT applied to patients with COVID-19 increased the 6-MWT distance (Δ: 40.13 m) [55]. These results indicate that IMT enhances respiratory muscle function, but its impact on exercise capacity varies depending on training duration, intensity, and the type of exercise testing. Our study contributes to this evidence by demonstrating a significant increase in 6-MWT distance using a standardized walking test. The training intensity was set at 50% of MIP. Based on the principle of specificity, high-intensity (50% of MIP), low-repetition training is typically prescribed to promote strength adaptations, whereas low-intensity (30% of MIP), high-repetition training is associated with endurance adaptations [56]. Lower intensities may therefore be insufficient to induce substantial strength gains, while substantially higher loads may reduce tolerability in symptomatic patients [24]. For these reasons, a moderate-to-high intensity (50% of MIP) was selected to specifically target improvements in inspiratory muscle strength in this population.

In this study, IMST increased functional exercise capacity by 96.44 m in post-COVID-19 patients with lung involvement. In the long term, high-intensity IMT has been shown to further increase 6-MWT distance in patients with heart failure [57]. Although Abodonya et al. [29] reported improvements in exercise capacity following IMT in post-acute patients immediately after mechanical ventilation weaning, their population differs from our cohort with post-COVID-19 syndrome. In addition, the 96.4 m improvement in 6-MWT distance observed in our study exceeds the clinically meaningful threshold previously reported in post-COVID-19 rehabilitation meta-analyses, where pooled effects of approximately 50 m were considered of clinical importance [54]. Increased respiratory muscle strength after training reduces respiratory muscle effort, enabling the ability to sustain higher exercise workloads [58]. Additionally, it reduces the perception of hyperpnea and dyspnea during exercise by enhancing breathing efficiency [59]. It may reduce the respiratory muscle metaboreflex and increase peripheral muscle oxygenation [16, 59]. Thus, increasing exercise capacity by reducing fatigue and leg fatigue during exercise. This integrated response—simultaneous improvements in respiratory efficiency, muscle oxygenation, and reduced perception of dyspnea—supports the hypothesis proposed in this study and aligns well with previous physiological models of the respiratory metaboreflex [15, 16]. In our study, we believe these physiological mechanisms contribute to improvements in patients’ functional exercise capacity following IMT. Our results provide important information to the literature by demonstrating that IMST improves functional exercise capacity in patients with post-COVID-19 syndrome and lung involvement.

Skeletal muscle abnormalities can be observed in both the acute and chronic phases of COVID-19 patients. Common problems include muscle weakness, exercise intolerance, and fatigue [9, 10]. To our knowledge, no study in the literature has investigated the effect of IMT applied alone on peripheral muscle strength in patients with post-COVID-19 syndrome and lung involvement. Hockele et al. investigated the effects of a pulmonary rehabilitation program that included IMT, aerobic, and resistance exercise training on handgrip strength, a marker of overall muscle strength, in patients with post-COVID-19 syndrome with lung involvement. It has been shown that patients’ grip strength in both hands increased after training [60]. Isolated IMT applied at 40% of MIP for 7 days a week, 6 weeks, has increased QF muscle strength in heart failure patients [17]. The present findings are therefore consistent with and extend these prior reports by isolating the specific effects of IMST on peripheral muscle adaptations, independent of concurrent aerobic or strength training.

In the current study, IMT increased patients’ peripheral muscle strength. In studies conducted in healthy individuals, prolonged inspiratory muscle contraction leads to metabolite accumulation and activates type IV phrenic afferents [61, 62]. This increase in metabolite concentration, known as the metaboreflex, results from increased ventilatory demand and causes diaphragm fatigue, as well as reducing blood flow to locomotor muscles through a systemic vasoconstrictor response in skeletal muscle [61]. Decreased blood flow to locomotor muscles may lead to reduced peripheral muscle strength. IMT has been suggested to increase peripheral muscle strength by improving inspiratory muscle function, reducing metaboreflex activity, and enhancing muscle blood flow [15, 16]. According to our results, IMST increased peripheral muscle strength and functional capacity in patients with post-COVID-19 syndrome and lung involvement. In this study, an increase in ΔSmO_2_, an indicator of increased QF blood flow, enhanced peripheral muscle strength by reducing respiratory muscle metaboreflex activity. These findings further support the hypothesis that IMST ensures systemic benefits by improving circulatory efficiency and oxygen distribution between respiratory and locomotor muscles. The effects of IMT on respiratory muscle metaboreflex activity should be investigated in future studies.

In patients with COVID-19, the amount of oxygen reaching the muscles decreases due to arterial hypoxemia caused by pulmonary system impairment, resulting in early lactate release and muscle fatigue [11, 12]. Impaired capillary blood flow following COVID-19 infection reduces the amount of oxygen delivered to the lungs and tissues, thereby causing tissue hypoxia [63]. In addition, the effects on mitochondrial functions may impair oxygen delivery to the muscles and decrease muscle oxygenation [11, 12]. Recent studies have also shown persistent impairments in exercise physiology and microvascular function after COVID-19, which may contribute to reduced oxygen utilization and functional capacity [11, 63, 64]. Our recently published study showed that in patients with post-COVID-19 syndrome and lung involvement, QF muscle deoxygenation was greater during maximal exercise than during submaximal exercise [65]. In another study, patients with post-COVID-19 syndrome with lung involvement were divided into two groups: those with mild and moderate functional limitations. Muscle oxygenation responses of the patients during submaximal exercise testing were examined. It was demonstrated that QF muscle oxygen saturations decreased similarly in both groups during submaximal exercise testing. Patients with moderate functional limitations walked shorter distances during submaximal exercise testing and had the same muscle oxygenation responses at lower workloads [66]. All this evidence suggests that muscle oxygenation may be impaired in patients after COVID-19 infection. For the first time in the literature, our study investigated the effects of IMST on muscle oxygenation in COVID-19 patients with lung involvement. These findings deepen our understanding of how peripheral oxygen utilization function and substantiate the current view that respiratory muscle training might help improve microvascular dysfunction after COVID-19.

In the present study, IMST increased resting muscle oxygen saturation of the QF muscle. During the submaximal exercise test, the change in muscle oxygen saturation decreased in the control group but remained unchanged in the IMST group. Muscle oxygen saturation change indicates the amount of oxygen used by the muscle [67]. In our study, IMT ensured that patients’ muscle oxygen saturation levels remained stable. The decrease in oxygen saturation in the control group suggests that peripheral muscle oxygenation may further decline in the future due to hypoxemia in patients with lung involvement who have had COVID-19. Additionally, the decrease in oxygen use by peripheral muscles can be prevented by IMT in these patients. In the current study, IMST increased resting muscle oxygen saturation. During the submaximal exercise test, the change in muscle oxygen saturation decreased in the control group but remained stable in the IMST group. This finding indicates that IMST helped sustain muscle oxygenation dynamics during the intervention period. Since no longitudinal follow-up was conducted, the findings suggest that the effects were maintained during the training period but require further investigation for long-term prevention.

These results suggest that IMST may maintain the balance between oxygen delivery and utilization during exercise, which is vital for preserving muscle endurance and delaying fatigue. However, it is also essential to consider the effect of subcutaneous adipose tissue thickness on NIRS signal penetration and accuracy, as increased adiposity can reduce light transmission and cause underestimation of muscle oxygenation levels. The same measurement protocol was used for all participants to reduce systematic bias.

Oxygen saturation is an indicator of oxygen delivery throughout the body. It is calculated from arterial blood, while SmO_2_ provides information about the amount of oxygen reaching and being used in the small vessels of the muscle. In our study, while SpO_2_ responses were normal during submaximal and maximal exercise tests, SmO_2_ values decreased in patients. In patients with post-COVID-19 syndrome with lung involvement, measuring peripheral oxygen saturation by pulse oximetry does not accurately detect hypoxia [65]. Therefore, exercise training programs should be designed by measuring muscle oxygenation during exercise, and future studies should examine the effects of exercise training on patients’ muscle oxygen responses.

In the current study, the majority of patients who had long-term symptoms prior to the training were inactive. In a study investigating the effects of a pulmonary rehabilitation program that included inspiratory muscle training on physical activity levels in patients with COVID-19, exercise training was found to increase physical activity more than inspiratory muscle training alone [68]. IMST did not improve physical activity levels in patients with post-COVID-19 syndrome and lung involvement. According to a published systematic review, physical activity counseling should be added to pulmonary rehabilitation programs, alongside exercise training, to improve physical activity duration in patients with COPD effectively [69]. Although IMST improves symptoms such as dyspnea and fatigue, counseling and education may help individuals gain physical activity habits. Therefore, combining IMST with behavioral interventions and physical activity counseling may result in greater long-term improvements in activity behavior and quality of life. Additionally, IMST enhanced physiological outcomes but did not increase physical activity levels. This may be due to behavioral or psychosocial barriers, such as fatigue, low motivation, or fear of effort, which are commonly reported after COVID-19 infection.

Dyspnea is one of the most common symptoms in long-term COVID-19 patients. Several studies have investigated the factors contributing to dyspnea and exercise intolerance, including impaired pulmonary gas exchange, hypoxemia, respiratory muscle weakness, and lactic acidosis [58, 70]. Our study is the first to explore the effect of IMST on dyspnea during daily activities in patients with post-COVID-19 syndrome and lung involvement. IMST decreased patients’ perception of dyspnea during domestic, physical, and total daily activities. Reducing dyspnea in post-COVID rehabilitation is clinically important, as it can directly improve daily functioning and participation in physical activities.

Exertional dyspnea occurs when the respiratory muscles are unable to handle the increased workload during exercise. The IMT alleviates dyspnea by providing resistance and loading the inspiratory muscles. It reduces dyspnea in patients with chronic obstructive pulmonary disease and interstitial lung disease [71, 72]. After IMT, improvements in patients’ breathing patterns and increased respiratory muscle strength help decrease shortness of breath. The mechanisms that improve dyspnea in COVID-19 patients have not yet been clarified. However, it is believed that IMST alleviates dyspnea by enhancing respiratory muscle function [73]. In patients with post-COVID-19 syndrome with lung involvement, IMST was an effective method to reduce dyspnea during daily living activities.

Fatigue affects physical function, activities of daily living, and quality of life in patients with COVID-19 and is highly prevalent among these patients [74–76]. Systematic reviews have reported post-infection fatigue prevalence in COVID-19 patients at 52% [77] and 64% [78], respectively. In our study, IMST reduced the severity of fatigue in patients with lung involvement and post-COVID-19 syndrome. According to the FSS, the IMST group’s fatigue perception decreased by 14.96 points, while the control group’s decreased by 1.15 points. After the training, the rate of severe fatigue in patients in the IMST group decreased from 85% to 45%, whereas in the control group it increased from 50% to 75%.

Hossein Pour et al. found that the perception of fatigue was reduced in the IMT group (40% of MIP, 30 min/day, 7 days/6 weeks) compared with the control group (10% of MIP, 30 min/day, 7 days/6 weeks) [79]. It was concluded that the increases in peripheral and respiratory muscle strength and functional exercise capacity achieved with IMT in heart failure patients may reduce perceived fatigue [17]. In our study, the observed improvements in respiratory and peripheral muscle strength and exercise capacity may have contributed to reducing fatigue perception. Reducing the perception of fatigue is crucial for patients to maintain greater independence in their daily lives. The IMST can be applied as a rehabilitation option in reducing fatigue, which is one of the most common debilitating symptoms in patients with post-COVID-19 syndrome.

In our study, the lack of direct measurement of adipose tissue thickness may have influenced muscle oxygenation measurements, as near-infrared spectroscopy–derived signals are affected by the skin and subcutaneous fat tissue surrounding the muscle. Although adipose tissue thickness was not directly measured, pre-training BMI values were similar between groups, and no significant changes in body weight were observed following the intervention, reducing the likelihood of systematic between-group bias. It should also be noted that, in addition to absolute SmO₂ values, changes in muscle oxygenation (ΔSmO₂) were analyzed to reflect time-dependent metabolic responses during exercise. Kinetic measurements reflecting time-dependent changes during exercise are considered more specific to muscle metabolic activity and may be less susceptible to the confounding effects of subcutaneous adipose tissue than absolute oxygenation levels [80]. Nevertheless, the absence of direct measurement of subcutaneous adipose tissue thickness remains a methodological limitation and should be addressed in future research. In addition, the IMST group received weekly supervised sessions, whereas the control group was followed up by phone. This difference in supervision may have introduced non-specific treatment effects, such as increased motivation or attention, which could have influenced the observed outcomes. Future studies may consider supervising the control group with a similar frequency and/or implementing a sham IMST protocol at a lower than therapeutic threshold to better control for attention-related or placebo effects. Outcomes were assessed immediately after the 8-week intervention period, as long-term follow-up was beyond the scope of the present study. Therefore, the sustainability of the observed improvements over time remains uncertain, and future research incorporating extended follow-up periods is warranted to evaluate potential maintenance, detraining effects, or symptom relapse. Furthermore, although physical activity was objectively monitored, no behavioral or lifestyle intervention was implemented. This may partly explain the absence of significant changes in activity-related outcomes.

Another limitation of the present study is that participants aged 75 years or older were excluded to minimize the potential confounding effects of age-related comorbidities and frailty and to ensure the safety and tolerability of the exercise protocol. As individuals over 75 years are commonly included in clinical practice and pulmonary rehabilitation programs, the generalizability of the findings to this older population may therefore be limited.

This study has several important strengths. It was designed as a randomized, controlled, triple-blind trial, which reduces potential bias. Adherence to the intervention was high, supporting the reliability of the findings. In addition, the study included comprehensive physiological and functional assessments, including objective measurements of muscle oxygenation, respiratory and peripheral muscle strength, and physical activity levels.

## Conclusions

The present findings support the use of isolated IMST in patients with post-COVID-19 syndrome and pulmonary involvement. In the present study, IMST led to improvements in functional exercise capacity, QF muscle oxygenation, and peripheral muscle strength in both upper and lower extremities, which served as primary outcomes in patients with post-COVID-19 syndrome with pulmonary involvement. In addition, it reduces dyspnea and perceived fatigue during daily activities. In our study, treatment compliance among patients was very high (95.71%). The addition of IMST, which has high patient compliance in treatment, to pulmonary rehabilitation programs may be beneficial in improving problems associated with long-term hypoxemia in patients with post-COVID-19 syndrome with pulmonary involvement. Our results indicate that IMST promotes comprehensive recovery by enhancing muscle function, improving oxygen utilization, and alleviating symptoms. 

## Data Availability

In the present study, all data are available on reasonable request from the corresponding author.

## Abbreviations

ATS: American Thoracic Society
COVID-19: Coronavirus disease 2019
CT: Computed tomography
ERS: European Respiratory Society
FSS: Fatigue severity scale
IMT: Inspiratory muscle training
IMST: Inspiratory muscle strength training
LCADL: London chest activities of daily living
METs: Metabolic equivalent
MIP: Maximal inspiratory pressure
MEP: Maximal expiratory pressure
QF: Quadriceps femoris
SmO_2_: Muscle oxygen saturation
THb: Total hemoglobin
6-MWT: Six-minute Walk test
